# An investigation of drug use among first-time arrestees from 25 county jails across the United States in 2023

**DOI:** 10.1186/s13722-025-00550-5

**Published:** 2025-03-07

**Authors:** Joseph E. Schumacher, Abdullah Ahsan, Amber H. Simpler, Adam P. Natoli, Bradley J. Cain

**Affiliations:** 1 NaphCare Charitable Foundation, Inc, 2101 Old Columbiana Road, Suite 100, Birmingham, AL 35216 USA; 2 Natoli Services, LLC, 1315 10th St, PO Box 1192, Huntsville, TX 77342 USA

**Keywords:** Drug use, Arrestees, Jails, Urine drug screen, Health care

## Abstract

**Background:**

Conducting research within a carceral health care context offers a unique view into the nature of drug use among arrestees with potential to identify and prevent drug use consequences. The purpose of this study was to characterize the nature and extent of drug use among first-time jail arrestees to inform detection and treatment.

**Methods:**

This study utilized a naturalistic research design to collect de-identified urine drug screens (UDS), jail characteristics, and arrestee demographic variables among arrestees indicating drug use from 25 jails across the United States in 2023 through a confidential data sharing agreement with NaphCare, Inc. using its proprietary electronic health record operating system. Descriptive statistics were used to detail the features of the dataset, Pearson’s chi-square tests of independence were performed to statistically analyze associations between UDS results and jail characteristics and arrestee demographics, and significant chi-square test results were further investigated by examining standardized residuals to clarify the nature and significance of within-group differences in proportions.

**Results:**

Of the 43,553 UDS cases comprising the final sample (28.8% of total arrestees), 74.8% (32,561) were positive for one or more drugs, and 25.2% of UDS cases were negative for all drugs. Among those who tested positive, 69.0% were positive for cannabis, 54.8% for stimulants, 29.6% for opioids, and 12.4% for sedatives. Arrestees were positive for multiple drugs half the time, with combinations of cannabis, stimulants, and opioids most common. Significant associations between drug use and both jail characteristics and arrestee demographics were found.

**Conclusions:**

Though drug use is not a recent phenomenon, the lethality potential of the drugs being used today is relatively new. Arrestees with positive urine drug screens are at heightened risk of adverse outcome due to sudden cessation of substance use. Findings highlight the need for objective clinical data to guide acute treatment of individuals at risk of withdrawing while detained.

While medicinal and recreational drug use in the United States has consistently occurred since the country’s inception [1], the evolving social acceptance, criminalization, and increased potency of select drugs have contributed to the nation’s current drug epidemic and carceral crisis [2–5]. Since President Nixon’s declaration of drug abuse as “public enemy number one” [6] and related adoption of the federal Comprehensive Drug Abuse Prevention and Control Act (Controlled Substances Act) of 1970 [7] and creation of the Drug Enforcement Administration (DEA) in 1973 [8], the “war on drugs” catalyzed the expansion of mass incarceration, thereby overfilling the nation’s correctional institutions with individuals convicted of non-violent, drug-related offenses. Each year more than 12 million Americans cycle in and out of jails with previous research focusing mostly on prisons limiting our understanding of the health, well-being, and healthcare utilization in jail settings [9]. In midyear 2022, jails held 663,100 persons in custody, 4% more than the year before [10].

Despite the imposition of mandatory sentencing and other strict drug laws, drug use has not been curtailed. Rather, the drug epidemic has morphed from the narcotics crisis of the 1950s to the rise of crack cocaine in the 1980s [11, 12] to today’s opioid epidemic (2000s-present) [13–15]. While the 2000s ushered in an era of decriminalization and legalization of certain drugs [16, 17], the nation continues to face record numbers of negative outcomes as it combats the “Fourth Wave” of the opioid overdose crisis [17] referencing the deaths related to combined illicit fentanyl and stimulant use. This crisis is most evident in this country’s jail population [18].

Drug use among individuals at the time of arrest and booking into a jail (hereafter “arrestees”) is high and significantly greater than use among the general population. According to the 2023 National Survey on Drug Use and Health (NSDUH), 61.8 million people or 21.8% of the general population age 12 or older used illicit drugs in the past year, with marijuana identified as the most frequently used drug [19]. Among arrestees from the National Institute of Justice (NIJ) Arrestee Drug Abuse Monitoring (ADAM) Programs [20, 21], estimated drug use prevalence between 1998 and 2003 was 6.4 million people, or approximately 65% of the arrestee population. In ADAM II (2007–2013), the most recent available report, the proportion of arrestees who tested positive for any drug ranged from 63% in Atlanta to 83% in Chicago and Sacramento [22]. Arrestees testing positive for multiple drugs ranged from 12% (Atlanta) to 50% (Sacramento). Marijuana remained the most frequently detected drug by urine testing, with prevalence estimates ranging from 34% (Atlanta) to 59% (Sacramento) [23]. Among ADAM II adult arrestees in the San Diego region, 77% of men and 75% of women tested positive for at least one drug, with methamphetamine and marijuana the most commonly detected drugs [24]. Similarly, researchers found prevalence estimates of substance use disorders (SUDs) were significantly greater among arrestees (63%) compared to US adults overall (5%) [25]. Moore and colleagues (2020) reported adults with lifetime drug-related legal problems were three to five times more likely to meet diagnostic criteria for a SUD, with stimulant use disorder being the most prevalent diagnosis [26].

Although researchers have highlighted differences between the general population and arrestees, research limitations may hinder knowledge about drug use patterns among arrestees. While the National Institute of Justice’s ADAM Programs [20, 21] revealed substance use patterns among jail detainees based on longitudinal drug use monitoring using bioassays in different geographical areas of the United States since 1987, this project concluded in 2013 and does not reflect the national opioid epidemic experienced over the past decade [20]. Also, ADAM II (2007–2013) was limited to a predominantly male sample across only 10 jails [27].

Studies of arrestees’ drug use based solely on self-report may be inherently biased [28–30]. While self-reporting is valuable when determining the circumstances surrounding drug use and diagnostic criteria [31], there are disincentives to admitting drug use upon arrest, which compromises the accuracy of self-reporting among jail arrestees [30]. Moreover, studies’ sample sizes, examination of jail characteristics, and means of determining drug use (e.g., self-report) tend to be limited in scope, and the effect of prior arrests on recent arrests was not typically considered in existing studies [32–35]. Each of these elements narrows the implications of previously published results. Most research on drug use and jail arrestees is independent of the clinical context in carceral settings, focusing instead on epidemiological, policy, or other research purposes with no deliberate emphasis on integrating findings into the jail health care system [10], even though drug-related fatalities are the third leading cause of death in United States jails [36]. Investigations addressing these limitations are necessary to understand drug use among arrestees adequately.

## Current study

Attempting to build upon past research on drug use among jail arrestees [37] this study includes the use of objective bioassay urine drug screens (UDSs) to measure common drugs of abuse and reporting on rates of combined drug use or testing positive for more than one drug. Drawn from 25 jails of varying size in three United States Census regions (West, South, Midwest), this study examined data from first-time male and female arrestees detained during the calendar year 2023 and examined associations between positive UDS results and jail characteristics, including size and location and arrestee demographics, including sex, race, and age.

Conducting research within this carceral health care context offers a unique estimation of the nature of drug use among arrestees with potential to identify and prevent acute drug use consequences such as drug withdrawal and overdose and inform clinical practice regarding withdrawal intervention. The purpose of this study was to characterize the nature and extent of positive UDS results, serving as a proxy for use of common drugs of abuse, among first-time jail arrestees to inform the detection, treatment, and prevention of adverse consequences associated with drug use by:

1. Describing and demonstrating the utilization of a national correctional health care provider and electronic health care records system to assess drug use among persons arrested and booked into municipal/county jails.
2. Reporting types and combinations of drug use (i.e., derived from point-of-care immunoassay testing) among first-time arrestees from 25 jails across the United States in 2023 and comparisons across jail characteristics and arrestee demographics.
3. Discussing the practical implications of assessing drug use among jail arrestees.

## Method

### Data source

Health data of individuals arrested and booked into jails (*arrestees*) across the United States were extracted from a proprietary electronic health record (EHR) system (TechCare 5.0^®^), de-identified, and provided to the authors under a confidential data-sharing agreement between NaphCare, Inc. (NaphCare) and NaphCare Charitable Foundation, Inc. (NCF). Because data were decoupled from identifiers (i.e., name, booking/medical record numbers, dates of birth, and social security numbers), Salus IRB (Case ID #23102-01) determined this archival project did not involve human subjects and would not require institutional review board (IRB) approval.

In 2023, NaphCare held contractual agreements to provide health care services in 30 jails, which are secure facilities operated under government authority to confine individuals accused of criminal offenses or convicted of minor/misdemeanor crimes. Of those facilities, 25 met the following inclusion criteria for this study: (1) had an active contract with NaphCare for the provision of comprehensive healthcare services throughout 2023; (2) conducted urine drug screening of arrestees self-reporting drug use or suspected of withdrawal risk and consented to a UDS; and (3) possessed complete results of UDSs documented in TechCare from 2023. One jail was excluded for not being fully operational during the year 2023, and four jails were excluded due to the implementation of a “test all” policy whereby all arrestees (i.e., not just individuals self-reporting drug use and believed to be at risk of withdrawal) were asked to submit to a UDS at the point of booking.

Study jails were located in three of the four primary United States Census Bureau census regions (West, Midwest, and South) [38]. Because only one jail was located in the Northeast, we elected to exclude this facility to avoid potential misrepresentation of geographic trends. Jails were also categorized by size based on contractual bed capacity (i.e., the number of individuals to be served by the contract and not the rated/design capacity of the facility). Size categories were adapted from the American Jail Association’s (n.d.) framework [39]: medium-large = 250–499 beds; large = 500–999 beds; and mega = 1,000 or more beds. Larger jails are typically located in and representative of more populated urban areas [40]. Table 1 shows the breakdown of study jails by geographic region and size.

**Table 1:** Frequency and percentages of study jails by size and geographical location

Jail Size	Jail Location							
	West		Midwest		South		All	
	*n*	%	*n*	%	*n*	%	*n*	%
Medium-Large	7	28	1	4	0	0	8	32
Large	4	16	2	8	4	16	10	40
Mega	1	4	2	8	4	16	7	28
All	12	48	5	20	8	8	25	100

Note. Geographical location of jails coded according to the census regions of the U.S. Census Bureau 2020) (Northeast not represented). Jail size categories based on the contracted bed capacity of a given facility: Medium-Large (250–499), Large (500–999), and Mega (≥ 1,000)

### Data extraction

Data were extracted from the EHR using Structured Query Language (SQL) scripts/queries, which included demographic and UDS information (when available) for all individuals booked into custody across 25 study jails from January 1 through December 31, 2023. Data were then exported as multiple. csv files from all sites and subsequently concatenated into two separate pandas DataFrames: one containing de-identified demographic information of all arrestees and one containing UDS results. Finally, Python and its libraries were used to compute descriptive statistics and frequencies and generate tables and figures.

### Drug withdrawal risk

All individuals arrested and booked into study jails in 2023 were counted as arrestees. At the point of booking, healthcare staff perform screening assessments to identify acute and/or chronic health concerns that may require triage and additional assessment. Of particular interest are individuals who may be chemically dependent and at risk for adverse outcomes due to withdrawal from select substances (e.g., alcohol, benzodiazepines, opioids), though the presence of other non-fatal withdrawal risk drugs (e.g., cannabis, stimulants) was anticipated. Arrestees determined to be at risk for withdrawal and possibly in need of detoxification were approached to submit a point-of-care urine sample for a UDS within 48 h of booking. Withdrawal risk was based on one or more of the following criteria: observation by health care or jail staff or arrestee’s self-report of intoxication, recent use of a psychogenic drug, misuse of addictive prescription medication, drug withdrawal symptoms, and/or history of medically assisted drug detoxification or related hospitalization.

All voluntarily provided UDS samples (UDS cases) were observed, collected, analyzed, and documented by registered or accredited on-site healthcare professionals. The extent of arrestees who falsely denied drug use or risk factors and/or declined to be tested could not be determined from available data, reflecting a study limitation and topic for future research (see Limitations and Future Directions section). Consequently, drug use was studied only among arrestees self-reporting drug use, believed to be at risk for withdrawal, possibly in need of detoxification, and who agreed to submit a sample for UDS within the context of this carceral healthcare delivery system.

### Sample selection

The study sample was comprised of individuals arrested in 2023 (i.e., the most recent full year of UDS data) with a UDS. To control for the influence of multiple arrests and repeated measures, individuals with an arrest history in the three prior years (2020–2022) were excluded from the study sample. For individuals with multiple arrests in 2023, only UDS results from their first arrest in 2023 were included. While it is recognized that individuals from this sample may have been arrested before this designated time epoch, 2020–2022 represents a time when described UDS procedures were consistently employed across the study jails and arrest data were reliable.

**Fig. 1:**
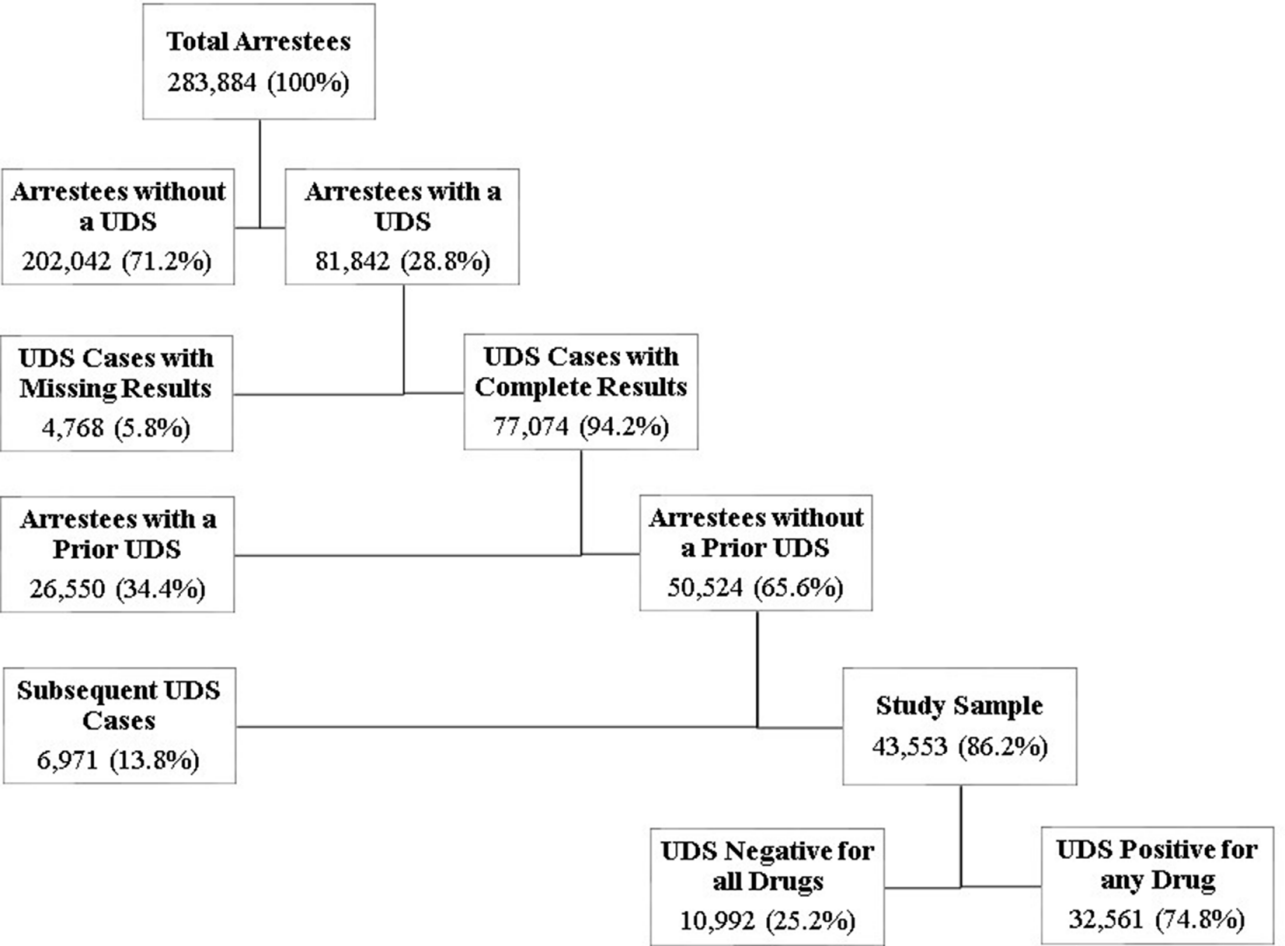
Study sample flowchart

The process of identifying first-time arrestees with a UDS and characterizing drug use among first-time UDS cases positive for any drug is described here and shown in Fig. 1. Arrestees from 2023 without a UDS were first removed from the sample. UDS cases with missing drug test results (no result for one or more of the 13 drugs tested) were then removed from the sample, as were arrestees with a prior booking in the past three years. If an arrestee was arrested multiple times in 2023, only their first arrest was used. The resultant study sample of first-time 2023 arrestees was partitioned into two groups: negative for all drugs and positive for any drug for further analysis of drug use variables.

### Urine drug screen (UDS)

Urine samples were screened for psychoactive drugs with the Quick Test Cup™ Multi-Drug Urine Cup (MD-U261) and the Rapid Test Strip, Urine, for fentanyl (FYL-U11) manufactured by 12 Panel NOW™. All 12 Panel NOW™ products are verified by Registrar Corp. 2022 U.S. FDA Registration. MD-U261 test results are negative or presumptive positive for the following drugs: opioid (OPI), methamphetamine (MET), amphetamine (AMP), benzodiazepine (BZO), cocaine (COC), methadone (MTD), oxycodone (OXY), marijuana (THC), barbiturates (BAR), buprenorphine (BUP), 3,4-Methyl enedioxy methamphetamine or “ecstasy” (MDMA), and phencyclidine (PCP) (Quick Test Cup™ insert). Separate FYL-U11 tested for fentanyl (FYL) (Rapid Test Strip, Urine insert).

UDS variables were derived from a negative or presumptive positive result for each of the 13 drugs (including FEN) tested. Preliminary inspection of raw data suggested cross-reactivity between AMP and MET, which led authors to consult with the forensic toxicologist at the Division of Workplace Programs, Center for Substance Abuse Prevention, Substance Abuse and Mental Health Services Administration (SAMHSA) (C. Lodico, personal communication, February 29, 2024) and the Vice President for Sales and Logistics at 12 Panel NOW™ (S. Zivanov, personal communication, February 22, 2024) about cross-reactivity concerns. Analyses of all UDS cases positive for AMP or MET (28,735) found that 93.5% were positive for both and 6.4% were positive for either. Based on these findings and the likelihood of cross-reactivity of presumptive positive MET and AMP expressed by these two experts, MET and AMP were conservatively combined to represent one category (i.e., referred to as MET/AMP). Differentiation between MET and non-MET amphetamines would require confirmatory testing, which is neither required nor feasible in jails and not necessary for the purpose the UDS is administered (i.e., identification of withdrawal risk arrestees for implementation of a detoxification protocol). Thus, findings from the resultant 12 drug types were utilized in this study.

UDS variables included *positive for any* of the 12 drugs tested versus *negative for all* drug types tested. The 12 drugs were then grouped into four drug types based on the nomenclature of the Diagnostic and Statistical Manual of Mental Disorders– Fifth Edition– Text Revision (DSM-5-TR) [41]: stimulants (COC, AMP/MET, MDMA); opioids (OPI, BUP, OXY, MTD, FEN); cannabis (THC); and sedatives (BZO, PCP, BAR). PCP, a dissociative anesthetic, and BAR, a central nervous depressant, were included in the sedatives (i.e., sedatives, hypnotics, and anxiolytics) group due to their sedating and hypnotic qualities [42, 43]. Finally, the number of presumptive positive drug test results from each drug type and positive for one or more drug types (combined or multiple drug use) were calculated.

### Statistical analyses

Frequencies and percentiles were calculated for UDS variables partitioned by jail characteristics and arrestee demographics and presented in numerical and graphic methods to summarize and describe the main features of the dataset. A series of Pearson’s chi-square tests of independence were performed to statistically analyze associations between UDS results and jail characteristics and arrestee demographics. Significant chi-square test results were probed by examining standardized residuals to clarify the nature and significance of within-group differences in proportions, with odds ratios calculated when possible (i.e., when examining arrestee sex). Standardized residuals greater than zero are interpreted to be more likely than expected under the null and those less than zero are less likely than expected under the null, with absolute values greater than 1.96 interpreted as significant.

## Results

### Study sample

Nearly one-third (81,842 or 28.8%) of all arrestees booked in 2023 were administered a UDS. Following the previously described exclusion rules, the final sample consisted of 43,553 first-time arrestees with complete UDS data (see Fig. 1). As shown in Table 2, UDS cases appeared evenly distributed across jail locations, with a greater number of UDS cases from large and mega-sized jails than medium-large jails. Consistent with prison populations of the United States, the proportion of males in the study sample was greater than females; however, the proportion of females in the current sample was greater than typically observed in United States jail populations [10]. Regarding arrestee race, White arrestees (52.2%) were mathematically more represented than Black arrestees (34.3%) with substantially less representation of other races, which is also generally consistent with national jail populations [10]. Similarly, young adults (age 20–39) were the most common age group at 60.3%, followed by adults (age 40–59) at 30.7%, seniors (age 60 and older) at 4.8%, and adolescents (age 15–19) at 4.2%.

### UDS positivity

Of the 43,553 UDS cases comprising the final sample, 74.8% were positive for one or more drugs and 25.2% of UDS cases were negative for all drugs. Presumptive positivity across all UDS cases for cannabis was 69.0%; 54.8% for stimulants; 29.6% for opioids; and 12.4% for sedatives. Descriptive statistics for UDS cases positive for any drug and negative for all drugs by jail characteristics and arrestee demographics are presented in Table 2 and separately by drug type in Table 3.

**Table 2:** Frequency and percentages of UDS cases and UDS results by jail characteristics and arrestee demographics

			UDS Result for Any Drug^b^			
	UDS Cases^a^ (*N* = 43553)		Positive (*n* = 32561)		Negative (*n* = 10992)	
Variable	*n*	%	*n*	%	*n*	%
Jail Characteristics						
Jail Location						
West	17,232	39.6	13,176	40.5	4056	36.9
South	13,904	31.9	10,241	31.5	3663	33.3
Midwest	12,417	28.5	9144	28.1	3273	29.8
Jail Size						
Medium-Large	5595	12.8	4322	13.3	5379	11.6
Large	20,406	46.9	15,027	46.2	1273	48.9
Mega	17,552	40.3	13,212	40.6	4340	39.5
Arrestee Demographics						
Sex						
Male	27,622	63.4	20,645	63.4	6977	63.5
Female	15,931	36.6	11,916	36.6	4015	36.5
Race						
White	22,716	52.2	17,745	54.5	4971	45.2
Black	14,937	34.3	10,917	33.5	4020	36.6
Hispanic	229	0.5	127	0.4	102	0.9
Asian	581	1.3	372	1.1	209	1.9
Other	76	0.2	55	0.2	21	0.2
Unknown	5014	11.5	3345	10.3	1669	15.2
Age Groups						
Adolescent (age 15–19)	1821	4.2	1399	4.3	422	3.8
Young Adult (age 20–39)	26,272	60.3	19,946	61.3	6326	57.6
Adult (age 40–59)	13,383	30.7	9905	30.4	3478	31.6
Senior (age 60 or older)	2077	4.8	1311	4.0	766	7.0

Note. Geographical location of jails coded according to the census regions of the U.S. Census Bureau 2020) (Northeast not represented). Jail size categories based on contracted bed capacity of a given facility: Medium-Large (250–499), Large (500–999), and Mega (≥ 1,000). Race Others include Indigenous, Hawaiian, and South Asian

^a^ UDS Case percentages were calculated by dividing the number of UDS cases for each jail characteristic and arrestee demographic variable by the total number of UDS cases

^b^ UDS Results percentages were calculated by dividing the number of UDS results (positive or negative) for each jail characteristic and arrestee demographic variable by the total number of UDS results (positive or negative)

**Table 3:** Frequency and percentages of positive UDS cases for drug type by jail characteristics and arrestee demographics

	Drug Type^a, b^								
	Any Drug (*n* = 32561)	Cannabis (*n* = 22464)		Opiates (*n* = 9632)		Sedatives (*n* = 4029)		Stimulants (*n* = 17850)	
Variable	*n*	*n*	%	*n*	%	*n*	%	*n*	%
Jail Characteristics									
Geographic Location									
West	13,176	8105	61.5	4510	34.2	1489	11.3	8293	62.9
South	10,241	7424	72.5	2789	27.2	1506	14.7	5236	51.1
Midwest	9144	6935	75.8	2333	25.5	1034	11.3	4321	47.3
Jail Size									
Medium-Large	4322	2655	61.4	1499	34.7	579	13.4	2767	64.0
Large	15,027	10,487	69.8	4386	29.2	1653	11.0	8209	54.6
Mega	13,212	9322	70.6	3747	28.4	1797	13.6	6874	52.0
Arrestee Demographics									
Sex									
Male	20,645	14,502	70.2	6264	30.3	2406	11.7	11,499	55.7
Female	11,916	7962	66.8	3368	28.3	1623	13.6	6351	53.3
Race									
White	17,745	10,694	60.3	6704	37.8	2636	14.9	11,724	66.1
Black	10,917	9202	84.3	1829	16.8	831	7.6	3948	36.2
Hispanic	127	76	59.8	33	26.0	20	15.7	69	54.3
Asian	372	221	59.4	85	22.8	43	11.6	243	65.3
Other	55	32	58.2	16	29.1	6	10.9	44	80.0
Unknown	3345	2239	66.9	965	28.8	493	14.7	1822	54.5
Age Group									
Adolescent (age 15–19)	1399	1322	94.5	177	12.7	91	6.5	264	18.9
Young Adult (age 20–39)	19,946	14,532	72.9	6091	30.5	2211	11.1	10,286	51.6
Adult (age 40–59)	9905	5892	59.5	2982	30.1	1468	14.8	6556	66.2
Senior (age 60 or older)	1311	718	54.8	382	29.1	259	19.8	744	56.8

Note. Geographical location of jails coded according to the census regions of the U.S. Census Bureau 2020) (Northeast not represented). Jail size categories based on contracted bed capacity of a given facility: Medium-Large (250–499), Large (500–999), and Mega (≥ 1,000). Race Others include Indigenous, Hawaiian, and South Asian

^a^ Frequency of positive UDS results for each drug type

^b^ Drug Type percentages were calculated by dividing the number of UDS results positive for a given drug type by the total number of positive UDS results for each jail characteristic and arrestee demographic variable. For example, UDS result positive for Cannabis in the West was calculated using the following formula: $\:\left(\frac{8105}{13176}\right)*100=61.5$

### Proportional differences in UDS positivity overall and by drug type

#### Jail characteristics

**Fig. 2:**
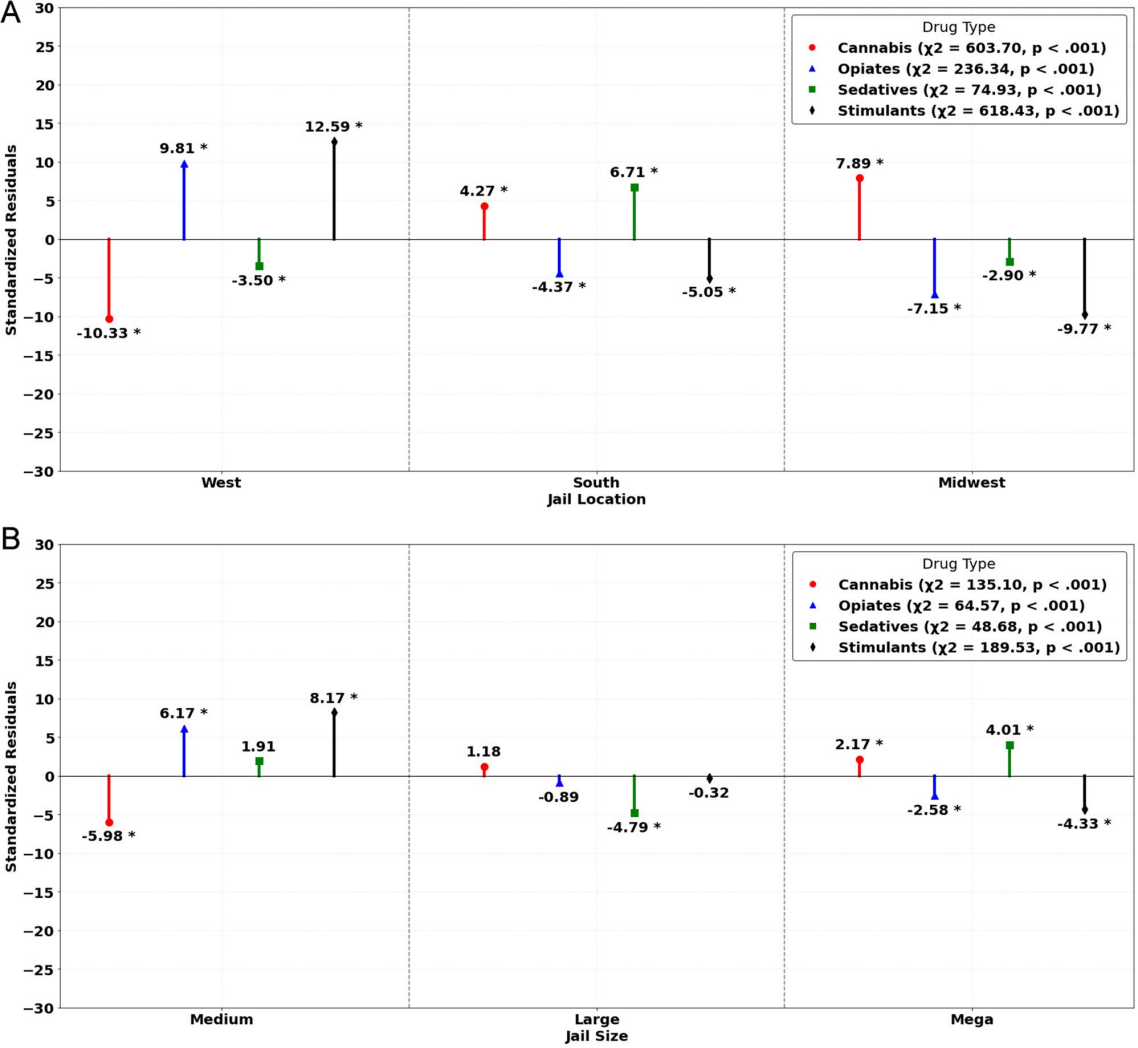
**a** Standardized residuals for jail location by drug type. **b** Standardized residuals for jail size by drug type. Note. Chi-square results reflect tests of association between UDS results for a given drug type and specified jail characteristics. Standardized residuals greater than zero are interpreted to be more likely than expected under the null and those less than zero are less likely than expected under the null. *Difference in proportions significant at *p* <.05 based on standardized residual

Although positivity rates were ostensibly similar across locations, statistical analysis revealed a significant association between jail location and UDS positivity, χ^2^_(2)_ = 43.70, *p* <.001 (see Table 4). The proportion of UDS results positive for any drug was significantly larger than expected in Western jails, while differences in proportions were not significant in Southern or Midwestern jails. Significant associations between jail location and positive UDS results were also observed separately for cannabis, opioids, sedatives, and stimulants with multiple significant differences in proportions for each of the three jail locations (see Fig. 2a).

Jail size was significantly related to overall UDS results, as well, χ^2^_(2)_ = 7,318.57, *p* <.001 (see Table 4). Medium jails yielded significantly fewer positive UDS results than expected and large jails yielded significantly more, though the proportion of positive UDS results in mega jails was not significantly different than expected under the null. As shown in Fig. 2b, there were significant associations between jail size and the proportion of UDS results positive for each of the four drug types (i.e., cannabis, opioids, sedatives, stimulants).

**Table 4:** Chi-square test of independence results testing associations between UDS results and jail characteristic or arrestee demographic

	Percentage of Arrestees in a Given Group with a Positive UDS Result				
	Any Drug	Cannabis	Opiates	Sedatives	Stimulants
Jail Characteristics					
Jail Location					
West	76.46*	61.51*	34.23*	11.30*	62.94*
South	73.66	72.49*	27.23*	14.71*	51.13*
Midwest	73.64	75.84*	25.51*	11.31*	47.26*
	*χ*^2^_(2)_ = 43.70	*χ*^2^_(2)_ = 603.70	*χ*^2^_(2)_ = 236.34	*χ*^2^_(2)_ = 74.93	*χ*^2^_(2)_ = 618.43
Jail Size					
Medium-Large	44.55*	61.43*	34.68*	13.40	64.02*
Large	92.19*	69.79	29.19	11.00*	54.63
Mega	75.27	70.56*	28.36*	13.60*	52.03*
	*χ*^2^_(2)_ = 7318.57	*χ*^2^_(2)_ = 135.10	*χ*^2^_(2)_ = 64.57	*χ*^2^_(2)_ = 48.68	*χ*^2^_(2)_ = 189.53
Arrestee Demographics					
Sex					
Female	74.80	66.82*	28.26*	13.62*	53.30*
Male	74.74	70.24*	30.34	11.65	55.70*
	*χ*^2^_(1)_ = 0.02, *p* =.896	*χ*^2^_(1)_ = 41.47	*χ*^2^_(1)_ = 15.65	*χ*^2^_(1)_ = 26.94	*χ*^2^_(1)_ = 17.58
Race					
White	78.12*	60.26*	37.78*	14.85*	66.07*
Black	72.99*	84.29*	16.75*	7.61*	36.16*
Hispanic	55.46*	59.84	25.98	15.75	54.33
Asian	64.03*	59.41*	22.85*	11.56	65.32*
Other	72.37	58.18	29.09	10.91	80.00*
Unknown	66.71*	66.94	28.85	14.74*	54.47
	*χ*^2^_(5)_ = 413.09	*χ*^2^_(5)_ = 1856.61	*χ*^2^_(5)_ = 1444.67	*χ*^2^_(5)_ = 347.97	*χ*^2^_(5)_ = 2471.63
Age in Years					
Adolescent (age 15–19)	76.83	94.50*	12.65*	6.50*	18.87*
Young Adult (age 20–39)	75.92*	72.86*	30.54*	11.08*	51.57*
Adult (age 40–59)	74.01	59.49*	30.11	14.82*	66.19*
Senior (age 60 or older)	63.12*	54.77*	29.14	19.76*	56.75
	*χ*^2^_(3)_ = 176.01	*χ*^2^_(3)_ = 1107.06	*χ*^2^_(3)_ = 202.67	*χ*^2^_(3)_ = 195.60	*χ*^2^_(3)_ = 1333.95

Note. All chi-square test statistics significant at *p* <.001 unless otherwise note. Geographical location of jails coded according to the census regions of the U.S. Census Bureau 2020) (Northeast not represented). Jail size categories based on contracted bed capacity of a given facility: Medium-Large (250–499), Large (500–999), and Mega (≥ 1,000). Race Others include: Indigenous, Hawaiian, and South Asian

*Difference in proportions significant at *p* <.05 based on standardized residual

#### Arrestee characteristics

**Fig. 3:**
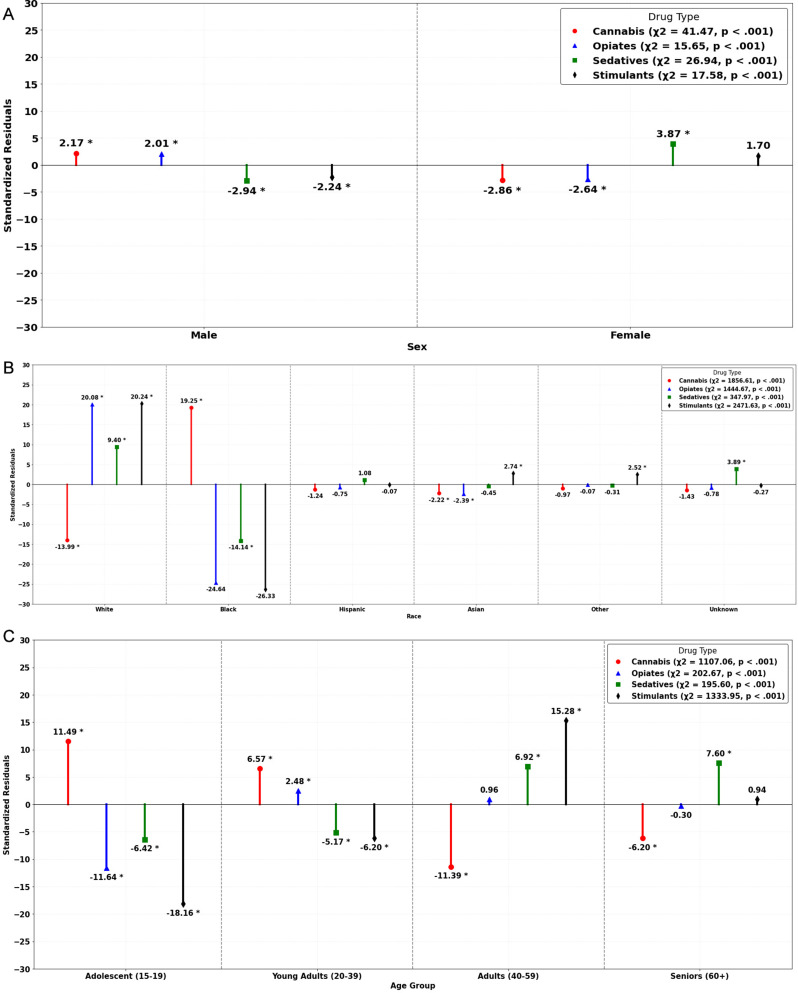
**a** Standardized residuals for arrestee sex by drug type. **b** Standardized residuals for arrestee race by drug type. **c** Standardized Residuals for Arrestee Age by Drug Type. *Note*. Chi-square results reflect tests of association between UDS results for a given drug type and specified arrestee demographic. Standardized residuals greater than zero are interpreted to be more likely than expected under the null and those less than zero are less likely than expected under the null. *Difference in proportions significant at *p* <.05 based on standardized residual

The proportion of positive UDS results among females was 0.748 and 0.747 among males, and the difference between these proportions was not significant, χ^2^_(1)_ = 0.02, *p* =.896 (see Table 4). However, there were several significant differences in the proportion of positive UDS results between males and females when UDS results for cannabis, opioids, sedatives, and stimulants were examined separately (see Fig. 3a). Based on odds ratios, the odds of a positive UDS result for a given drug coming from a female arrestee compared to a male arrestee were 14.7% lower for cannabis (OR = 0.85; 95% CI [0.81, 0.90]), 9.5% lower for opioids (OR = 0.90; 95% CI [0.86, 0.95]), and 9.2% lower for stimulants (OR = 0.91; 95% CI [0.87, 0.95]). Conversely, the odds of a UDS positive for a sedative were 1.20 (95% CI [1.12, 1.28]) times higher if the arrestee being tested were female than if the arrestee being tested were male.

There was a significant association between arrestee race and the proportion of positive UDS testing results, χ^2^_(5)_ = 413.09, *p* <.001(see Table 4). Generally, significantly more positive UDS results than expected were observed among White arrestees whereas the opposite was observed for arrestees who were Black, Hispanic, Asian, or Unknown, and no significant difference was observed among arrestees included in the Other Race category. Significant proportional differences were also found individually for cannabis, opioids, sedatives, and stimulants (see Fig. 3b). White and Asian arrestees produced significantly fewer positive UDS results for cannabis than expected if there was no effect of race, whereas Black arrestees produced significantly more. Regarding opiates, significantly more positive UDS results than expected were produced by White arrestees and the opposite was observed among Black and Asian arrestees. The proportion of UDS results positive for sedatives was significantly smaller than expected among Black arrestees and significantly larger than expected for White arrestees and arrestees for whom race was unknown. Finally, White, Asian, and Other Race arrestees produced significantly more positive UDS results for stimulants than expected under the null, whereas the proportion of UDS tests positive for stimulants was significantly smaller than expected among Black arrestees.

Age was also significantly associated with positive UDS results, *χ*^2^_(3)_ = 176.01, *p* <.001 (see Table 4). As depicted in Fig. 3c, multiple patterns between age group and drug type emerged: adolescent and young adult arrestees produced significantly more positive UDS results than expected for cannabis, while adult and senior arrestees produced significantly fewer positive results for cannabis than expected. The opposite was observed for sedatives and partially observed for stimulants, with a significantly higher probability of positive UDS results among adult and senior arrestees for sedatives and stimulants, whereas significantly fewer positive UDS results than expected for both sedatives and stimulants were observed among adolescents and young adults. Lastly, the proportion of positive UDS results for opioids was significantly smaller than expected for adolescents and larger than expected for young adults, though no significant differences in proportions were observed among adult or senior arrestees.

### Drug use combinations

Finally, positivity rates across UDS cases were examined to differentiate arrestees based on the number of drug types detected and distinct combinations of two to four drugs (see Table 5). Positivity for only one drug type was found in approximately half (51.1%) of the UDS cases with cannabis (63.3% of single drug cases) being the most common drug type, followed by stimulants (25.6% of single drug cases). Opioids (7.6% of single-drug cases) and sedatives (3.5% of single-drug cases) were the least common single-drug type. The remaining UDS cases were positive for multiple drug types, with 34.2% of UDS cases positive for two drug types, 12.4% positive for three drug types, and 2.3% of UDS cases positive for all four drug types. Of UDS cases positive for two drug types, over half (53.0%) were positive for cannabis and stimulants and roughly one-quarter (25.1%) were positive for opioids and stimulants, with the four remaining combinations occurring infrequently (i.e., *≤* 10.4%). UDS cases positive for three drug types made up 12.4% of all positive UDS cases, with the combination of cannabis, stimulants, and opioids occurring most frequently. The positivity of all four drug types occurred in only 2.3% of positive UDS cases.

**Table 5:** Frequency of positive UDS cases for drug type combinations of one to four drugs rank ordered from most to least prevalent

Number of Drugs^a^	Rank Order^b^					
	1st	2nd	3rd	4th	5th	6th
One Drug *n* = 16,651 (51.1%)	Cannabis	Stimulants	Opiates	Sedatives		
*n*	10,541	4261	1266	583		
%	63.3	25.6	7.6	3.5		
Two Drugs *n* = 11,137 (34.2%)	Cannabis, Stimulants	Opiates, Stimulants	Cannabis, Opiates	Cannabis, Sedatives	Sedatives, Stimulants	Opiates, Sedatives
*n*	5897	2798	1157	627	398	260
%	53.0	25.1	10.4	5.6	3.6	2.3
Three Drugs *n* = 4,042 (12.4%)	Cannabis, Opiates, Stimulants	Cannabis, Sedatives, Stimulants	Opiates, Sedatives, Stimulants	Cannabis, Opiates, Sedatives		
*n*	2612	622	531	277		
%	64.6	15.4	13.1	6.9		
Four Drugs *n* = 731 (2.3%)	Cannabis, Opiates, Sedatives, Stimulants					
*n*	731					
%	100					

^a^ Percentages for combination groups were calculated by dividing the number of UDS cases positive for a given number of drugs by the total number of positive UDS cases (*n* = 32561). For example, the following formula was used to calculate the percentage of UDS cases positive for only one drug: $\:\left(\frac{16651}{32561}\right)*100=51.1.$

^b^ Percentages for each specific drug type combination in each row (one, two, three, or four drugs) were calculated by dividing the number of UDS cases positive for a given drug combination by the total number of cases producing UDS results positive for the corresponding number of drugs. For example, the following formula was used to calculate the percentage of UDS cases positive for only cannabis: $\:\left(\frac{10541}{16651}\right)*100=63.3$

**Fig. 4:**
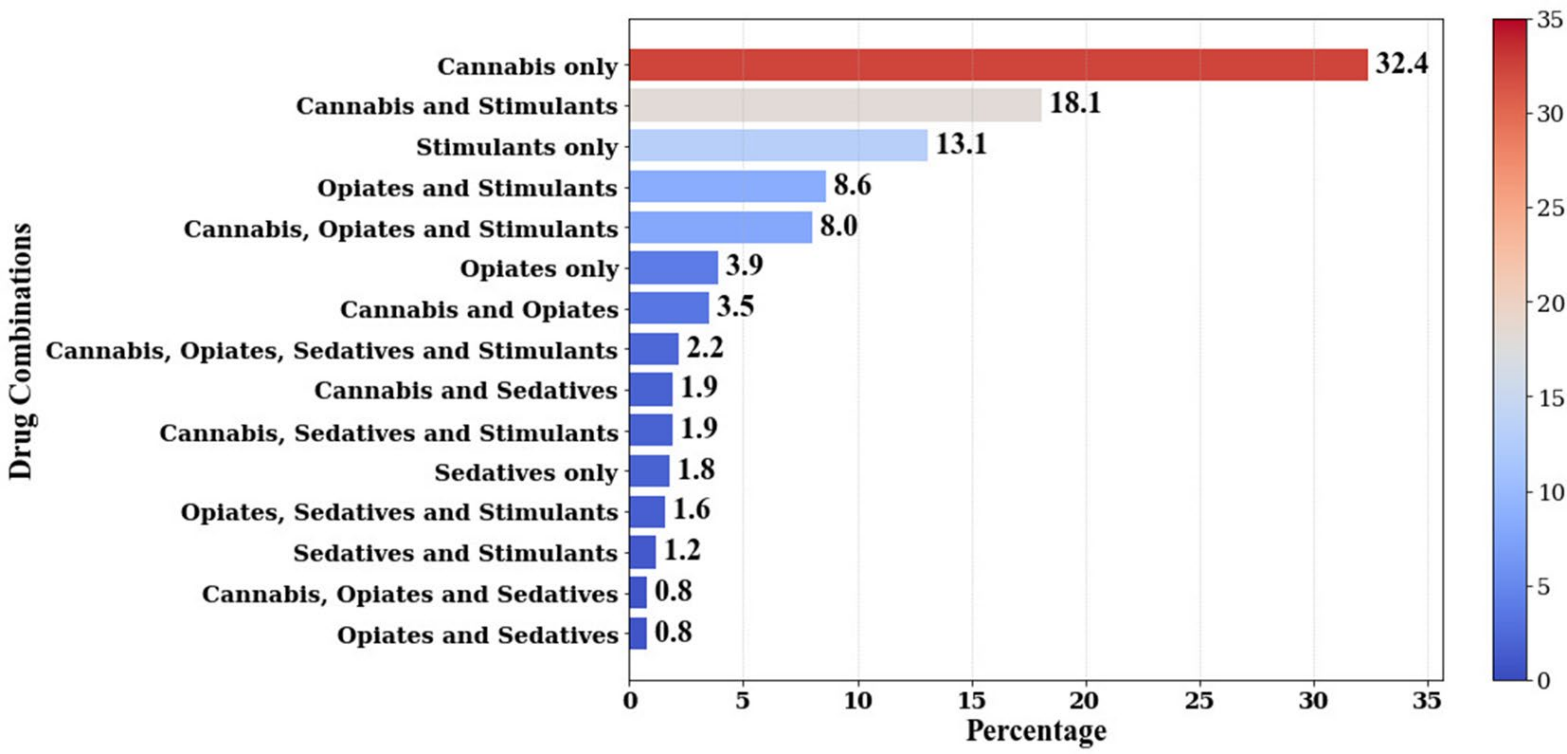
Percentage of positive UDS cases by drug type and combinations of drugs

Figure 4 illustrates the frequency with which each drug type and multiple drug combinations occurred among the positive UDS results in the study sample, ordered from most to least frequent. Findings revealed cannabis alone was the most common positive UDS result, accounting for almost one-third (32.4%) of all positive UDS cases. The combination of cannabis and stimulants, accounting for 18.1% of positive UDS cases, emerged as the second most common multiple-drug pattern. Stimulants alone were detected in 13.1% of cases, followed by the drug combination of opioids and stimulants (8.6%), then the combination of cannabis, opioids, and stimulants (8.0%). All other drug use combinations were observed in less than 4% of positive UDS cases, with positive UDS cases due to sedatives alone accounting for 1.8% of positive UDS cases. The least common drug combinations involved opioids and sedatives, either alone or in combination with cannabis. The positivity of all four drug types falls in the middle of the rankings at 2.2%.

## Discussion

This investigation of UDS results among first-time arrestees in county jails across the United States analyzed a unique sample of arrestees who were screened and tested for drug use in a healthcare context based on the determination of withdrawal risk and voluntariness. Despite certain limitations in this selection process and reliance on routinely collected healthcare data, discussed in detail below, the reported findings are useful reflections of the prevalence rates and patterns of drug use as they are known to correctional healthcare providers (i.e., as they are documented in EHRs). This research also offers a unique opportunity to consider the current findings in the context of prior research on drug use in the general population and epidemiological jail studies.

While 21.9% of the general population were estimated to have used drugs in the past year [19] and a range of 63–83% in jail settings were positive for drugs [22], almost one-third (28.8%) of arrestees in this study were deemed at risk for drug withdrawal and 74.8% of those tested positive for any drug. One can see that, although not operationally identical, the current results roughly correspond with these prior studies. Cannabis (marijuana) and stimulants were the most common drug types detected in the sample, which is consistent with the literature [24], and almost half of those testing positive for any drug tested positive for multiple drugs [23].

Geographic patterns of positive UDS results among arrestees in this study were inversely related between jail locations in the West and South, with a greater likelihood of cannabis and stimulants in the West; a greater likelihood of cannabis and sedatives in the South; and with predominate use of cannabis in the Midwest. The higher rate of positive UDS results in the West, mostly cannabis and stimulants, was consistent with the findings of the ADAM II project in Sacramento and San Diego [24]. The greater likelihood of testing positive for opioids and stimulants in medium-sized jails and more cannabis and sedatives in mega jails is new to the literature and may reflect emerging trends in rural (medium) versus urban (mega) jurisdictions not previously captured by large-scale research projects. Future research can explore this more directly.

Among the arrestee demographic findings, males and females did not differ in overall drug positivity, consistent with ADAM II in San Diego [24]; however, findings from this study revealed sex differences when analyzed at the drug type level. Female arrestees were less likely to test positive for cannabis, opioids, and stimulants than males and more likely to use sedatives. This finding is possibly linked to greater use of medications [44, 45] for psychiatric conditions [46, 47] among females, a hypothesis to be tested in future studies. White arrestees were more likely to test positive overall and for sedatives, opioids, and stimulants than other races while Black arrestees consenting to UDS were more likely to test positive for cannabis. Regarding age, younger arrestees (i.e., adolescents and young adults) were more likely to test positive for cannabis but less likely to test positive for other drug types. Adults were at the greatest risk for UDS results indicating the use of sedatives and stimulants, while seniors were at a greater risk of results positive for sedatives. Adolescents were less likely than expected to test positive for opioids, sedatives, or stimulants. Combined, and in the context of voluntary UDS, findings suggest ethnicity, race, and age may be important cultural and demographic factors to incorporate into a model of drug use assessment and treatment [48, 49] in this population.

Finally, this study found half of the arrestees undergoing UDS were positive for more than one drug. Combinations of cannabis and stimulants, opioids and stimulants, and cannabis, opioids, and stimulants were the most common, with stimulants intriguingly present in each combination. Additionally, stimulants and opioids were rarely positive alone and, rather, were more commonly detected in various combinations with other drug types. This could be explained by the practice of “speedballing,” or combining stimulants and opioids to experience the intense high of the stimulant while offsetting the negative effects with an opioid (or cannabis), or the unintentional use of an opioid, such as fentanyl while using stimulants, which could result in dangerous consequences such as heart failure, overdose, or death.

### Limitations and future directions

This study addresses certain concerns with existing research of drug use among arrestees by analyzing a sample of arrestees in 2023, including both males and females, utilizing objective immunoassay point-of-care measures of drug use, relying on a larger and more representative sample of disparate jail locations and jail sizes across the United States, examining drug use at the level of individual drugs and an exploration of multiple drug use patterns, and characterizing drug use within a naturalistic healthcare context to inform screening, treatment, and prevention of negative drug-related outcomes including overdose and death in jails. Despite these myriad strengths, the limitations of the present study should be considered.

First, results cannot be interpreted as reflecting accurate population rates of types of drug use as might be found in an epidemiological or prevalence estimate study. Arrestees’ data used for this study were obtained within a healthcare delivery context in jails and not from a random or proportion/stratified sampling procedure. As such, the findings are limited to a subsample of arrestees who were identified to be at risk for negative drug use consequences determined by self-report and/or observation and who voluntarily submitted to a UDS, thereby excluding arrestees reluctant to disclose or endorse drug use risk criteria due to concerns about stigma, confidentiality, retribution, or other reasons. Despite this, the findings from this study characterize a real-life clinical context in jails and likely *underestimate* the actual extent of the problem. Strategies and policies designed to more accurately, economically, and confidentially detect drug use among all arrestees should be investigated. Additionally, individuals with prior arrests were excluded to focus on first-time arrestees, which prevented analysis of the impact of repeat arrests or changes in UDS results over time.

Second, drugs of abuse only, not alcohol, were analyzed for this study. Alcohol is likely the most prominent intoxicant among arrestees [50] and is associated with serious - even fatal - withdrawal symptoms [51]. Although there is value in understanding trends in alcohol intoxication among arrestees, we omitted this research question because observed or self-reported use of alcohol in this database was not consistent or reliable enough to confidently analyze, and archived immunoassay test results did not include alcohol. Given the prevalence of alcohol use and the importance of identifying arrestees at risk for alcohol withdrawal symptoms and the relative ease of alcohol testing, future research should focus on reliable alcohol use assessment, including saliva or breathalyzer tests to ascertain blood alcohol content and alcohol withdrawal risk measures to prevent negative alcohol-withdrawal related outcomes in jail.

Third, the assessment of drug types used was determined by point-of-care immunoassay tests only. Point of care or screening tests are designed to test for the absence of a drug or a presumptive presence or positivity of a drug and fail to provide additional discriminatory information. For example, UDS testing does not discriminate between illicit or legitimately prescribed drugs, discern inadvertent use of a drug (e.g., adulterated substances), provide a quantitative measure, measure prolonged use or tolerance, detect brief drug use or metabolized drugs, or meet SUD criteria for diagnosis. Furthermore, UDS cannot determine whether drugs were taken simultaneously or within the same use session, as some substances remain detectable in urine for days after use. However, research on people who use drugs and drug surveillance data indicate that drug combinations are common. Despite this, it is important to acknowledge this limitation when interpreting the findings. Moreover, while UDS is effective at detecting common drugs of abuse, it may not identify novel psychoactive substances emerging in the unregulated drug market, including certain fentanyl analogs [52]. Policies and investigation of the practical implementation of both quantitative and qualitative assessment strategies to provide sufficient information for an informed treatment response are needed.

Fourth, while UDS tests used for this study were designed to detect unique metabolites of specific drugs with unique reagents, a presumptive positive outcome is likely to lack specificity and risk cross-reactivity between drugs tested than a negative result. We found significant cross-reactivity between amphetamine and methamphetamine, necessitating combining the two drugs. Point-of-care immunoassay testing is still an economic and practical detection strategy that would likely be enhanced by the addition of confidential structured self-report techniques, questions of incremental validity and utility for future studies to examine.

Fifth, for the first look at these data, drugs were grouped into four types (cannabis, opiates, sedatives, and stimulants) instead of looking at each drug separately for ease of descriptive and statistical comparisons by jail characteristics and arrestee demographics. While the message is still clear and the findings useful, future investigations of specific drugs and drug combinations, like fentanyl and methamphetamine, may provide more sensitive and specific information about their unique contribution and impact on withdrawal management, maintenance treatment, and overdose risk.

Finally, while the exclusion of individuals with arrests from 2020 to 2022 was intended to control for multiple arrests, this period coincided with significant shifts in law enforcement practices, court operations, and crime trends [53]. The pandemic led to changes in arrest patterns, including decreases in drug-related offenses and property crimes due to altered policing strategies, as well as increases in substance use and domestic violence. Given these factors, it is possible that the 2023 first-time arrestee population differs from pre-pandemic cohorts in ways that were not explicitly considered.

## Conclusion

Jails play a critical role in addressing drug use within communities by providing healthcare services that include screening, assessment, withdrawal management, and in-house treatment programs. The UDS data used in this study exist because correctional healthcare administrators and clinical professionals recognize the urgent need to identify and treat individuals at risk for adverse withdrawal-related outcomes. To mitigate these risks, the jails in this study have implemented protocols to screen all incoming arrestees. However, the current system has limitations—many individuals may go undetected due to reliance on self-report data and the voluntary nature of UDS sample submission. While we do not advocate for compulsory UDS testing, objective clinical data enhance clinicians’ ability to accurately identify and treat those at risk of withdrawal, ultimately improving care outcomes.

Jails can serve as vital healthcare touchpoints by implementing evidence-based interventions such as medication for opioid use disorder (MOUD), standardized withdrawal management, and routine substance use screening. Harm reduction strategies, including naloxone distribution and overdose prevention education, further reduce risks upon release. Access to peer recovery support, behavioral health counseling, and reentry care coordination ensures continuity of treatment, helping individuals transition to community-based services. By integrating these approaches, jails can improve health outcomes, reduce overdose deaths, and serve as a critical entry point for substance use treatment.

The lethality of today’s drug supply, particularly with the rise of fentanyl and other high-potency substances, has transformed drug use from a long-standing concern into an urgent public health crisis—one that is particularly pronounced within correctional populations. Jails, as critical points of healthcare contact for justice-involved individuals, are uniquely positioned to implement evidence-based interventions that reduce harm and improve health outcomes. The findings from this study suggest that enhanced detection and intervention efforts could benefit a significant proportion of first-time arrestees, as well as many others cycling through the criminal legal system. Strengthening jail-based screening and treatment programs could not only prevent withdrawal-related complications but also serve as a pivotal entry point for broader substance use treatment and harm reduction strategies in the community.

## Data Availability

Data were de-identified and provided to the authors under a confidential data sharing agreement between NaphCare, Inc. (NaphCare) and NaphCare Charitable Foundation, Inc. (NCF) and so are not publicly available.

## Abbreviations

AMP: Amphetamine
BAR: Barbiturate
BZO: Benzodiazepine
BUP: Buprenorphine
COC: Cocaine
EHR: Electronic Health Record
FYL: Fentanyl
IRB: Institutional Review Board
MDMA: Ecstasy
MET: Methamphetamine
MTD: Methadone
NaphCare: NaphCare, Inc
OPI: Opiate
OXY: Oxycodone
PCP: Phencyclidine
SQL: Structured Query Language
TechCare: TechCare 5.0
THC: Marijuana, Cannabis
UDS: Urine Drug Screen
